# Best Practice Framework of Fracture Liaison Services in Spain and their coordination with Primary Care

**DOI:** 10.1007/s11657-020-0693-z

**Published:** 2020-04-25

**Authors:** A. Naranjo, S. Ojeda, M. Giner, M. Balcells-Oliver, L. Canals, J. M. Cancio, E. Duaso, J. Mora-Fernández, C. Pablos, A. González, B. Lladó, F. J. Olmo, M. J. Montoya, A. Menéndez, D. Prieto-Alhambra

**Affiliations:** 1 Department of Rheumatology, Doctor Negrin University Hospital, Las Palmas de Gran Canaria, Spain; 2 Bone Metabolism Unit, Department of Internal Medicine, “Virgen Macarena” University Hospital, Seville, Spain; 3 Amgen, Barcelona, Spain; 4 Geriatrics Service, Centre Sociosanitari El Carme, Badalona Serveis Assistencials (BSA), Barcelona, Spain; 5 Geriatrics Service, Igualada Hospital, Barcelona, Spain; 6 Geriatrics Service, Coordinator FLS Hospital Clínico San Carlos, Madrid, Spain; 7 Geriatrics Service, Complejo Asistencial Universitario Salamanca, Salamanca, Spain; 8 Fracture Liaison Service, Hospital Son Llatzer, Mallorca, Spain; 9 Fracture Liaison Service, “Virgen Macarena” University Hospital, Seville, Spain; 10 Fracture Liaison Service, Hospital Vital Álvarez-Buylla, Asturias, Spain; 11 Nuffield Department of Orthopaedics, Rheumatology and Musculoskeletal Sciences (NDORMS), University of Oxford, Oxford, UK

**Keywords:** Fragility fracture, Secondary prevention, FLS, Fracture Liaison Service, Best Practice Framework

## Abstract

***Summary*:**

The coordination of Fracture Liaison Services (FLS) with Primary Care (PC) is necessary for the continuity of care of patients with fragility fractures. This study proposes a Best Practice Framework (BPF) and performance indicators for the implementation and follow-up of FLS-PC coordination in clinical practice in Spain.

**Purpose:**

To develop a BPF for the coordination of FLS with PC in Spain and to improve the continuity of care for patients with fragility fractures.

**Methods:**

A Steering Committee selected experts from seven Spanish FLS and related PC doctors and nurses to participate in a best practice workshop. Selection criteria were an active FLS with an identified champion and prior contact with PC centres linked to the hospital. The main aim of the workshop was to review current FLS practices in Spain and their integration with PC. A BPF document with processes, tools, roles, and metrics was then generated.

**Results:**

Spanish FLS consists of a multidisciplinary team of physicians/nurses but with low participation of other professionals and PC staff. Evaluation and treatment strategies are widely variable. Four desired standards were agreed upon: (1) Effective channels for FLS-PC communication; (2) minimum contents of an FLS clinical report and its delivery to PC; (3) adherence monitoring 3 months after FLS baseline visit; and (4) follow-up by PC. Proposed key performance indicators are (a) number of FLS-PC communications, including consensus protocols; (b) confirmation FLS report received by PC; (c) medical/nursing PC appointment after FLS report received; and (d) number of training sessions in PC.

**Conclusions:**

The BPF provides a comprehensive approach for FLS-PC coordination in Spain, to promote the continuity of care in patients with fragility fractures and improve secondary prevention. The implementation of BPF recommendations and performance indicator tracking will benchmark best FLS practices in the future.

**Electronic supplementary material:**

The online version of this article (10.1007/s11657-020-0693-z) contains supplementary material, which is available to authorized users.

## Introduction

Osteoporosis and its associated fragility fractures are globally common conditions contributing significantly to morbidity, mortality, and healthcare spending, and thus constitute a major public health problem [1].

According to recent statistics from the International Osteoporosis Foundation, worldwide, 1 in 3 women and 1 in 5 men over the age of 50 will experience fragility fractures in their lifetime [2]. In Spain, it was estimated in 2013 that a total of 552,879 women and 161,922 men would suffer fragility fractures in the next 10 years [3].

Patients experiencing the first fragility fracture are at a significantly higher risk of subsequent fractures [4]. Peri- and postmenopausal women with low trauma fracture have approximately twice the risk of subsequent fractures compared with similar patients without fracture [5]. Moreover, osteoporosis treatments are more cost-effective when prescribed to older adults with prior fracture [6]. Thus, clinicians caring for fracture patients must consider options for secondary fracture prevention. In patients at moderate fracture risk, bone mineral density can help guide therapeutic decisions. In interventional studies aimed at improving osteoporosis management after a fracture, bone densitometry was used in a median of 43% of patients [7]. However, in Spain, the use of bone densitometry (Dual energy X-ray absorciometry) for fracture risk assessment is limited [8]. Moreover, despite the availability of medications proven to reduce the risk of further fractures [9], fewer than 20% of individuals who sustain a fragility fracture receive such therapies within the first year following the fracture [10, 11]. This results in a pervasive worldwide treatment and strategy gap for secondary fracture prevention [11, 12]. There are multiple contributors to this large gap, such as clinicians failing to adhere to treatment guidelines [11], the low priority assigned to secondary fracture prevention by primary care (PC) and hospital physicians [13], and poor patient adherence to treatment [11].

Faced with this situation, in 2011, the Fracture Working Group of the Committee of Scientific Advisors of the International Osteoporosis Foundation (IOF) published a position paper on coordinator-based systems for secondary prevention in fragility fracture patients. The paper consolidated knowledge on the development, effectiveness, and common factors that underpin successful clinical systems designed to close the secondary fracture prevention care gap [14]. Fracture Liaison Services (FLSs) are care coordinator-based secondary fracture prevention programmes that systematically identify fragility fracture patients, then assess, investigate, and treat them for underlying osteoporosis as appropriate [15]. They are a cost-effective strategy for reducing the osteoporosis care gap, refracture rate, and mortality [16, 17]. A nurse coordinator or other professional acts as liaison between the patient, hospital team, and PC physician, to ensure continuity of care [13]. If effective communication between the FLS and PC is established, PC physicians are well placed and willing to manage osteoporosis care in the longer term [12].

However, in the current scenario in Spain, once the fracture has healed, there is no clear reference as to who should undertake the patient’s subsequent follow-up and care [18]. Existing models for secondary fracture prevention mostly come from Anglo-Saxon countries [14, 19–21] and must be adapted to the specific local healthcare environment [18].

The aim of this project was to develop a Best Practice Framework (BPF) for FLS-PC coordination in Spain, to guarantee the continuity of care for patients with fragility fractures.

## Methods

The Spanish Society for Research on Bone and Mineral Metabolism (SEIOMM) supported a workshop to define and standardise processes, tools, roles, and metrics for FLS-PC coordination.

A literature review regarding current FLS practices in Spain was performed to inform the workshop. Searches were made in international (PubMed) and national databases (MEDES, IBECS), as well as grey literature (research published in non-commercial form, e.g. reports, conference proceedings, or doctoral theses/dissertations), up until December 2017. The search strategy focused on the pathology of interest (“osteoporosis”), fracture as the main complication, and treatment management in FLS (Supplementary Table 1).

A Steering Committee was created with champions from three FLS centres in Spain and the UK (Hospital Dr. Negrín, *n* = 2; NDORMS, *n* = 1). Champions (*n* = 9) and case managers (*n* = 1) from seven consolidated Spanish FLS were invited to participate in a workshop, together with their related PC doctors (*n* = 11) and nurses (*n* = 8) (Supplementary Table 2). Selection criteria for FLS participation were to have an active FLS, with a well-identified champion, and to have prior contact with the PC centres linked to the hospital.

During the workshop, each champion presented current practices and connection model with PC in their healthcare area. The FLS best practices followed by the Steering Committee, and information derived from the literature review, were presented as key indicators of the performance and coordination of the FLS with PC.

Two discussion groups were requested to reach a consensus on the relationship between FLS and PC. One group focused on the communication between the FLS and PC, to ensure the clinical report is complete and reaches the PC centre. The second group focused on the coordination of PC doctors and nurses when they receive the clinical report, and coordination with the hospital for patient follow-up. During the first round of discussions, the Steering Committee posed questions to define best practice standards and how they should be measured. During the second round, conclusions from each discussion group were debated. The experts then generated the first draft of the BPF, reviewed the draft, made suggestions, and approved the final version of the BPF for FLS-PC coordination in Spain. The BPF, which includes the recommendations proposed and performance indicators, is presented below.

## Results

### Current practices in Spanish FLS

Current practices in Spanish FLS and their coordination with PC are presented (Table 1), considering a Spanish excellence FLS (FLS of the Hospital Dr. Negrín, a national reference with vast expertise) and the other seven FLS participating in the study (Supplementary Table 2).

**Table 1:** Current actions performed in Spanish FLS

Patient identification							
Emergency list	Patient lists from hospital services			Orthopaedics	Geriatrics	Plaster room	Functional spinal unit
	Rheumatology	Orthogeriatrics	PC				
75.0%	37.5%	37.5%	37.5%	25.0%	25.0%	25.0%	25.0%
Evaluation							
Blood test	Densitometry	Spine X-ray	FRAX	Nutritional assessment	Fall risk scale	TBS	Functional capacity assessment
100%	87.5%	75.0%	37.5%	37.5%	37.5%	25.0%	12.5%
Intervention							
Pharmacological treatment	Nutritional advice	Lifestyle recommendations	Calcium and vit D recommendations	Fall prevention	Gait rehabilitation	Physical exercise	Occupational therapy
100%	87.5%	75.0%	75.0%	62.5%	50.0%	37.5%	25.0%
Follow-up							
Hospital visit			PC physician	Phone		Electronic prescription platform	
75.0%			62.5%	37.5%		12.5%	
Coordination with PC							
Clinical report delivery to PC	Pathway for regular communication with PC		Periodic training sessions in PC			Liaison person	
75.0%	75.0%		25%			25%	

*FRAX* fracture risk assessment tool, *TBS* trabecular bone score

#### FLS composition and patient identification

**Fig. 1:**
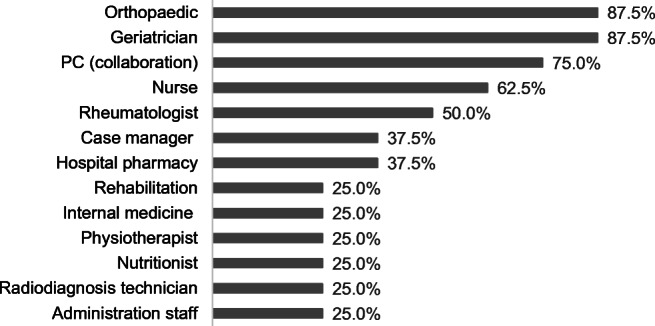
FLS composition

FLS consist of a multidisciplinary team, mainly comprised by physicians from different specialties and nurses (Fig. 1). Each FLS unit identifies patients with fragility fracture through several pathways. The main one is the emergency list, while other less common modes include patient lists from rheumatology services, orthogeriatrics, or PC.

#### Type of fractures included

All FLS include hip fracture, and most of them include vertebral fracture, and fracture of the radius and proximal humerus.

#### Evaluation

All FLS request a blood test to evaluate the patient’s condition. Most of them also collect densitometry and an X-ray of the spine. Data collected to a lesser extent are Fracture Risk Assessment Tool (FRAX), nutritional assessment, fall risk scale, trabecular bone score, or assessment of functional capacity.

#### Intervention

The FLS follow different scientific criteria to determine the intervention and prescription recommendations for secondary fracture prevention, including the NOF [22] and SEIOMM [23] criteria. All FLS recommend pharmacological treatment when appropriate; and most of them give nutritional advice; make recommendations about lifestyle and calcium and vitamin D supplements; or include a fall prevention service. Recommendations on gait rehabilitation, physical exercise, or occupational therapy are less common.

#### Follow-up

In most FLS (75.0%), patient follow-up is undertaken at hospital visits face-to-face with the specialist (orthopaedics, geriatricians, rheumatologists, and internal medicine physicians). Time to the first follow-up visit varies among centres, ranging from 1 month to 2 years, as well as the number of visits carried out, from none to three in a year. In 37.5% of FLS, general follow-up is also performed by the PC physician, while in 25.0% of FLS, follow-up is exclusively performed in PC.

In some FLS, the patient is also monitored by telephone to assess treatment adherence, and only the Spanish excellence FLS also monitors treatment adherence through an electronic prescription platform.

### Coordination with PC

Three-quarters of FLS have an established pathway for continuous coordination with PC, whether through e-mail/telephone/fax or virtual consultations. However, a designated individual to manage this coordination is available only in 25.0% of FLS, being a support technician or a case manager nurse.

Another means of communication with PC is the clinical report sent to the PC physician. In 83.3% of the cases, the report is sent directly to the PC physician or delivered via the patient, and in 33.3% of cases, the report is shared by common software used by the hospital and PC.

Only 25.0% of FLS deliver training sessions at PC centres to emphasise the importance of secondary prevention, treatment adherence, and understanding the report issued by the FLS.

### BPF standards for the coordination of FLS with PC in Spain

Four standards were agreed upon: (1) Effective communication channels between the FLS and PC; (2) minimum contents of the FLS clinical report and its delivery to PC; (3) treatment adherence 3 months after the visit to FLS; and (4) follow-up by PC doctor and nurse. Performance indicators proposed were (a) number of FLS-PC communications, including consensus protocols; (b) confirmation of the FLS clinical report reception by PC; (c) medical and nursing appointments in PC after the FLS report; and (d) training sessions received in PC (Table 2).

**Table 2:** Recommendations for hospital FLS-PC coordination and performance indicators

Standard	Recommendation	Performance indicator
1. FLS-PC communication procedure Effective FLS-PC communication allows PC to obtain clarification about specific cases or doubts regarding the recommendations issued by FLS and maintain the intervention proposed, ensuring continuity of care for fragility fracture patients	Means of communication: • Consultant from the FLS: on-site (periodic visit to PC centre) or virtual (online) • Email address available for consultations • Regular meetings (quarterly) in the PC centres • Development of consensus protocols (referral, treatment) with PC • Rotation of PC physicians and nurses in the FLS • Training sessions for health professionals in PC by FLS members, with the participation of PC physicians • Promotion of the detection of fragility fracture (including vertebral fracture) in PC: medical and nursing medical record	• Number of on-site and virtual consultations, e-mails sent and doubts resolved, meetings, protocols created, rotations carried out, and fractures identified by PC • Number of training sessions for health professionals held in PC
2. FLS clinical reports The FLS generates a clinical report at patient discharge, which is sent to the PC physician	Minimum data to include in the clinical report: • Patient affiliation (personal data and medical history), previous fracture, current fracture, future fracture risk (DXA and FRAX with DXA), blood analysis, and Spinal X-ray (if performed) • Previous treatment, renal function, comorbidities, other, i.e. previous adverse effect, glucocorticoids • Pharmacological and non-pharmacological recommendations	• Number of reports generated by the FLS and percentage received by PC • Percentage of reports with minimum data
3. Adherence control by the FLS Adherence to treatment should be confirmed after the baseline visit	Adherence should be confirmed by the FLS in the first 3 months, by both telephone call and electronically, and documented in one of the following: • FLS database • PC medical history by the PC doctor/nurse	• Percentage of patients contacted for adherence in the first 3 months, calculated from the total number of patients with indication of treatment attended in the FLS • Percentage of adherent patients in the first 3 months, calculated from the total number of patients with indication of treatment attended in the FLS
4. Patient follow-up by PC doctors and/or nurse Follow-up should be performed within 6 months of receipt of the FLS report in PC and promoted through a training plan. The PC nurse and/or PC physician would be responsible of the follow-up	Means of follow-up: • Establish an automatic alert when the report is received in PC: appointment with the doctor and the nurse • Educational workshops for patients: development of homogeneous and basic material for patients, incorporation of the PC physicians, FLS members, PC nurses and physiotherapists in the training sessions	• Percentage of patients with a follow-up for fracture in medical record (physician or nurse) in the first 6 months • Number of training educational workshops for patients in PC

*DXA* bone densitometry, *FRAX* fracture risk assessment tool

## Discussion

The osteoporosis care gap after fragility fractures is growing substantially. The reason this care gap exists, and persists, is multifactorial in nature [15]. Lack of clarity regarding where clinical responsibility lies has been identified as a major contributing factor [24]. Although initiating treatment in the orthopaedic department and then delegating care to PC physicians has been recommended [25], simply delegating care to PC does not positively influence provision of appropriate preventative measures nor does it improve subsequent fracture prevention. This is because neither orthopaedic surgeons who treat acute fractures nor PC physicians who provide long-term healthcare appear to be engaged in secondary prevention [26, 27]. It is therefore important to motivate PC physicians to become more involved in the management of low-impact fractures [26]. International recommendations for secondary fracture prevention advocate that processes should be in place to ensure reliable provision of long-term management of fracture risk. In healthcare systems with established PC infrastructure, local PC must be involved in developing the processes that they will implement for post-fracture care [15].

It is encouraging that Spain is actively creating FLS for secondary fracture prevention [28]. Spanish FLS participating in this study share common characteristics and patterns, such as their multidisciplinary composition, type of fractures treated, and main pathways for fracture-patient identification. However, they differ significantly in the extent of evaluation, outreach of intervention strategies, and the frequency and routes of patient follow-up. Moreover, several improvement opportunities exist in the coordination with PC.

As previously described, the implementation of FLS may be limited by a lack of PC participation [26]. In this context, standardised practices are needed to achieve effective coordination between newly created FLS and PC, and thus improve the continuity of care of patients with fragility fractures.

In general, there is limited information regarding how the coordination between FLS and PC is performed and little data about the performance of this communication. Chang et al. [29] identified several treatment gaps in current FLS and provided recommendations for best practice establishment of future FLS across the Asia-Pacific region. Their findings emphasise the importance of PC physicians continuing to prescribe treatment and ensure service remains convenient. In current practice worldwide, specialists rely on PC to manage osteoporosis. However, PC doctors routinely do so only if advised by specialists, and osteoporosis experts—usually endocrinologists or rheumatologists—have no reason to interact with the patient post-fracture [28]. The involvement of other specialists, such as geriatricians, in the acute care of elderly patients with fractures (mainly hip fractures) in “Orthogeriatric Units”, has shown to improve secondary fracture prevention [30]. Another proven solution to close the secondary fracture prevention care gap is to establish an FLS [15], necessarily coordinated with PC, highlighting the need for the development of a BPF for this coordination.

In the present study, four BPF standards were proposed to address the main needs for FLS-PC coordination in Spain: (1) Promotion of FLS-AP communication, (2) unification of FLS clinical report metrics, (3) systematic control of the adherence to treatment by the FLS, and (4) improvement of patient follow-up by PC.

The four standards we propose have previously been reported as relevant issues in osteoporosis management. The FLS coordinator, who takes care of all aspects of the process (patient identification, investigation, and therapeutic intervention), is responsible for providing adequate medical information to PC physicians. FLS experts proposed seven possible general communication channels, ranging from face-to-face meetings to telecommunication channels, such as the telephone or e-mail, in line with general literature regarding communication systems in healthcare [31]. An important secondary fracture prevention strategy relies on the FLS report to PC with treatment recommendations [7]. Our BPF recommends which items to include in this report and possible ways to deliver it to PC. Medication adherence remains a particular challenge in osteoporosis [32], with little consensus on how to identify non-adherent patients [33–35]. Our BPF recommends checking patient adherence to treatment in the first 3–4 months after the indication of treatment, by both telephone call and electronic receipt.

For BPF implementation in the seven FLS and their PC centres participating in the project, a training plan for PC will be designed. It will encompass basic information (osteoporosis, secondary prevention) and the BPF standards. Training sessions will be delivered by the champion of the FLS and a defined case manager of the PC centre. In this regard, providing PC physicians with information on fracture risk, osteoporosis, and appropriate preventative measures in discharge reports has proven to be an important part of secondary fracture prevention [17]. Afterwards, a case report form will be designed according to the metrics established in the workshop to monitor the implementation and performance of the BPF standards. The performance indicators will be recorded quarterly by the FLS champion and nurse/case manager of each FLS and PC centre.

Our proposed BPF will serve as a model for the creation of new FLS and as a guide for improving existing ones. Adherence to the recommendations in our BPF may improve the management and follow-up of patients with fragility fracture. Overall, the percentage of patients suffering from a new fracture could be reduced and, therefore, the direct and indirect costs associated with the fragility fracture lowered.

## Limitations of the study

The study was conducted from the perspective of Spanish healthcare professionals and the results might not be extrapolated to other countries. However, the development of standards for the coordination FLS-PC in Spain is crucial, given the increasing number of FLS created, and the results of the study provide a guide for the optimal long-term management of patients with fragility fractures. Further follow-up must be undertaken to demonstrate the efficiency of the BPF in increasing the compliance and long-term rate of therapy utilisation.

## Conclusions

Local and national prevention strategies must be put in place rapidly to reverse the increasing number of fragility fractures occurring in Spain. The BPF provides a comprehensive approach for the coordination between FLS and PC in Spain, to promote the continuity of care in patients with fragility fractures and improve secondary prevention. The implementation of the BPF in clinical practice will provide feedback for ongoing improvement of BPF standards and to benchmark the best FLS in the future.

## Electronic supplementary material

ESM 1 (DOCX 15 kb).

ESM 2 (DOCX 13 kb).
