# Supplementary material for: Best Practice Framework of Fracture Liaison Services in Spain and their coordination with Primary Care
Source: Arch Osteoporos. 2020 Apr 25;15(1):63. doi: 10.1007/s11657-020-0693-z (PMC7183494; doi:10.1007/s11657-020-0693-z)
Supplement: Supplementary file 1 — (DOCX 15 kb). [file 11657_2020_693_MOESM1_ESM.docx]

**Supplementary Table 1.** List of search terms in PubMed, MEDES and IBECS

| N. | Terms related to the pathology |
| --- | --- |
| #1 | "osteoporosis” [Mesh/All fields] |
| N. | **Terms related to the complications** |
| #2 | “fragility fracture” [All Fields] |
| #3 | “osteoporotic fractures” [Mesh/All fields] |
| #4 | "fractures, Spontaneous" [Mesh/All fields] |
| #5 | "secondary Prevention" [Mesh/All fields] |
| #6 | "preventive Health Services" [Mesh/All fields] |
| #7 | “prevention” [All Fields] |
| N. | **Terms related to treatment management** |
| #9 | "fracture Liaison Service” [All fields] |
| #10 | "FLS" [All fields] |
| #11 | “care network” [All Fields] |
| #12 | “multidisciplinary unit” [All Fields] |
| #13 | “multidisciplinary team” [All Fields] |
| #14 | "fracture unit" [All fields] |
| #15 | “fracture clinic” [All fields] |
| #16 | “osteoporosis clinic” [All Fields] |
