# Supplementary material for: Best Practice Framework of Fracture Liaison Services in Spain and their coordination with Primary Care
Source: Arch Osteoporos. 2020 Apr 25;15(1):63. doi: 10.1007/s11657-020-0693-z (PMC7183494; doi:10.1007/s11657-020-0693-z)
Supplement: Supplementary file 2 — (DOCX 13 kb). [file 11657_2020_693_MOESM2_ESM.docx]

**Supplementary Table 2.** Participating FLS

**Steering Committee**

| FLS Hospital Dr. Negrín, Las Palmas de Gran Canaria, Canarias |
| --- |
| FLS Oxfordshire (UK) |

**Healthcare areas**

| FLS Anoia. Hospital d´ Igualada, Barcelona |
| --- |
| FLS Hospital Son Llàtzer, Palma de Mallorca, Mallorca |
| FLS Hospital Universitario Virgen Macarena, Sevilla |
| FLS Hospital Clínico San Carlos, Madrid |
| FLS Complejo Asistencial Universitario Salamanca, Salamanca. |
| FLS Hospital Vital Álvarez Buylla, Mieres, Asturias |
| FLS Centre Sociosanitari El Carme. Badalona Serveis Assistencials (BSA), Barcelona |
